# Supplementary material for: Design of Protegrin-1 Analogs with Improved Antibacterial Selectivity
Source: Pharmaceutics. 2023 Jul 30;15(8):2047. doi: 10.3390/pharmaceutics15082047 (PMC10458893; doi:10.3390/pharmaceutics15082047)
Supplement: Supplementary file 1 [file pharmaceutics-15-02047-s001.zip › pharmaceutics-2521557-supplementary.pdf]

pharmaceutics

MDPI

Supplementary Materials: Design of Protegrin-1 Analogs with Improved Antibacterial Selectivity

Ilia A. Bolosov [1], Pavel V. Panteleev [1], Sergei V. Sychev [1], Veronika A. Khokhlova [1], Victoria N. Safronova [1], Ilia Yu. Toropygin [2], Tatiana I. Kombarova [3], Olga V. Korobova [3], Eugenia S. Pereskokova [3], Alexander I. Borzilov [3], Tatiana V. Ovchinnikova [1,4] and Sergey V. Balandin [1,*]

[1] M. M. Shemyakin & Yu. A. Ovchinnikov Institute of Bioorganic Chemistry, the Russian Academy of Sciences, 117997 Moscow, Russia; bolosov@ibch.ru (I.A.B.); p.v.panteleev@gmail.com (P.V.P.); ovch@ibch.ru (T.V.O.)
[2] V. N. Orekhovich Research Institute of Biomedical Chemistry, 119121 Moscow, Russia
[3] State Research Center for Applied Microbiology & Biotechnology (SRCAMB), 142279 Obolensk, Russia
[4] Department of Biotechnology, I. M. Sechenov First Moscow State Medical University, 119991 Moscow, Russia
* Correspondence: arenicin@mail.ru

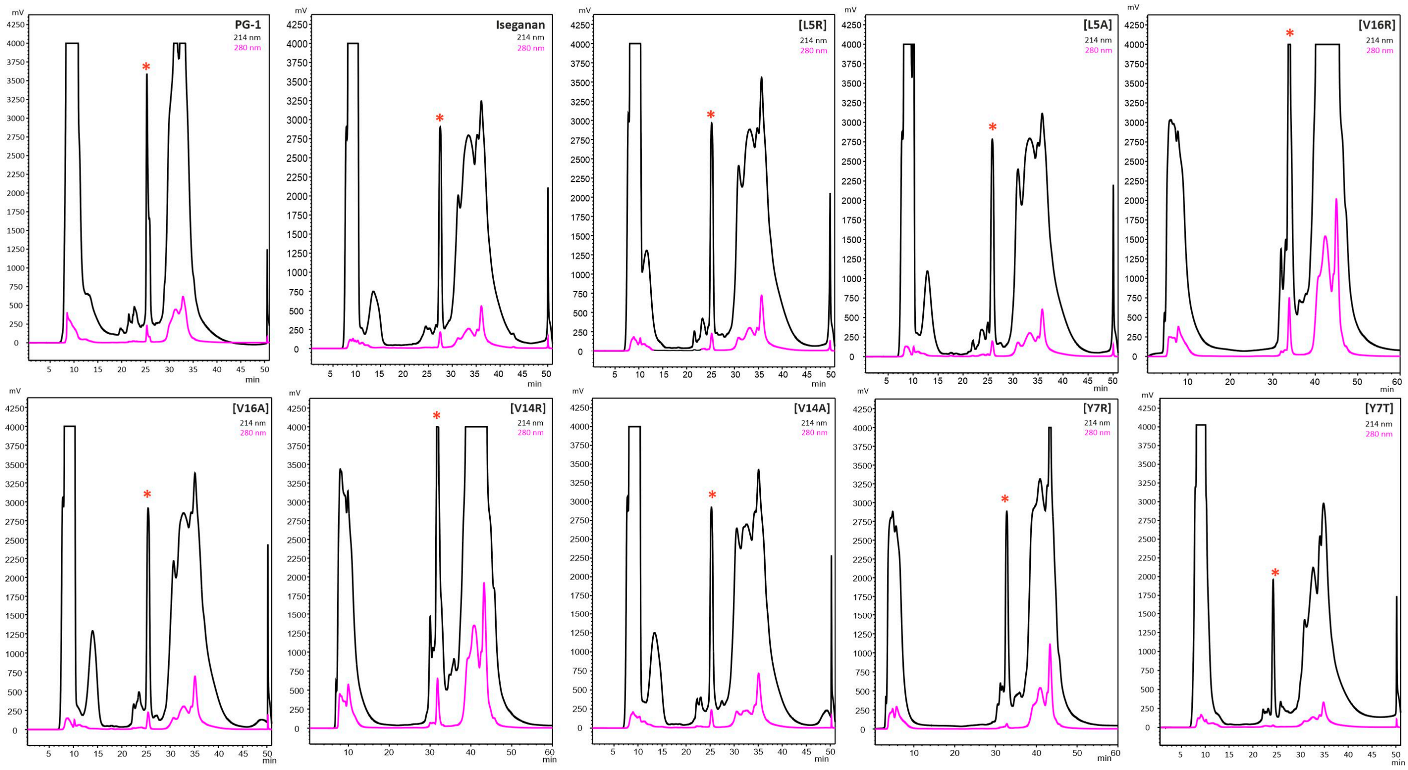

Figure S1. Reversed-phase high-performance liquid chromatography (RP-HPLC) purification of the recombinant protegrin analogs. The fractions of the target peptides are marked with an asterisk.

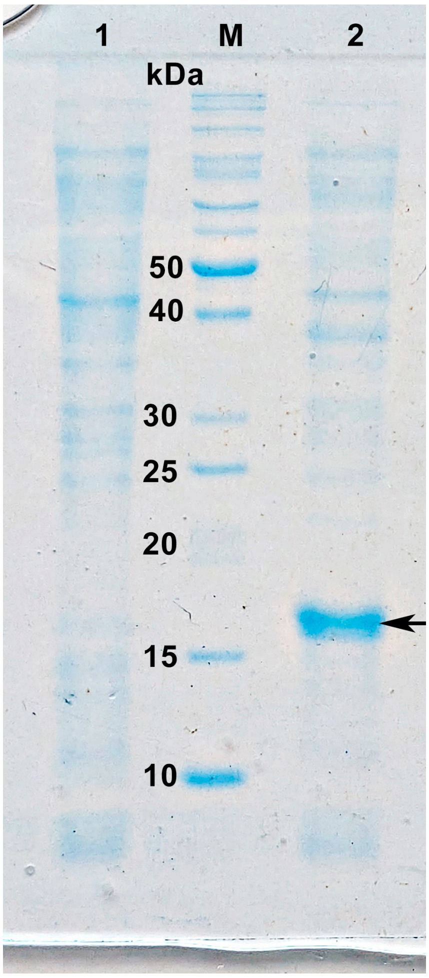

Figure S2. SDS-PAGE of the total lysate of E. coli BL21 (DE3) cells before (1) and after (2) IPTG induction, with β-mercaptoethanol. M – molecular mass standard. The arrows point at the target fusion protein (15.7 kDa), containing the [V16R] analog.
